# A novel technique for pediatric femoral locked submuscular plate removal: the ‘push-pull’ technique

**DOI:** 10.1186/1749-799X-8-21

**Published:** 2013-07-11

**Authors:** Martin F Hoffmann, John Gburek, Clifford B Jones

**Affiliations:** 1 Grand Rapids Medical Education Partners, 1000 Monroe Ave NW, Grand Rapids, MI 49503, USA; 2 College of Human Medicine, Michigan State University, 15 Michigan Street NE, Grand Rapids, MI 49503, USA; 3 Orthopaedic Associates of Michigan, 230 Michigan Street NE, Grand Rapids, MI 49503, USA

## Abstract

Submuscular and minimally invasive plate insertion is gaining popularity reducing the need for large open approaches and resulting in a smaller operative ‘footprint.’ With pediatric fractures and titanium implants, fibrous and osseous ingrowth to the implant and osseous implant integration may interfere with implant removal. Therefore, the small minimally invasive implant insertion procedure may require a large maximally invasive exposure for implant removal after fracture healing. To reduce soft tissue damage, bleeding, scarring, and pain associated with implant removal, a minimally invasive procedure utilizing the pre-existing incisions while controlling the implant is efficient and beneficial. The surgical technique is described, and a case series of 21 treated pediatric femoral fractures illustrates the successful performance of the procedure and its limitations.

## Background

Locked plating is very popular with increasing locked plating indications [1]. The main indications of locked plating are poor bone quality, fracture comminution and/or bone loss, and short segment fixation [2,3]. Additionally, submuscular and minimally invasive plate insertion is gaining popularity reducing the need for large open approaches, interfragmentary fixation, and bone grafting [4-8], and resulting in smaller, more cosmetically appearing scars [9]. Bridge plating with or without locked screws utilizes balanced fixation relying on longer plates and selective screw insertion leaving many screw hole sites empty. With plate irritation, patient preference, or surgeon preference, removal of submuscular plates may become necessary [10-15].

**Figure 1:**
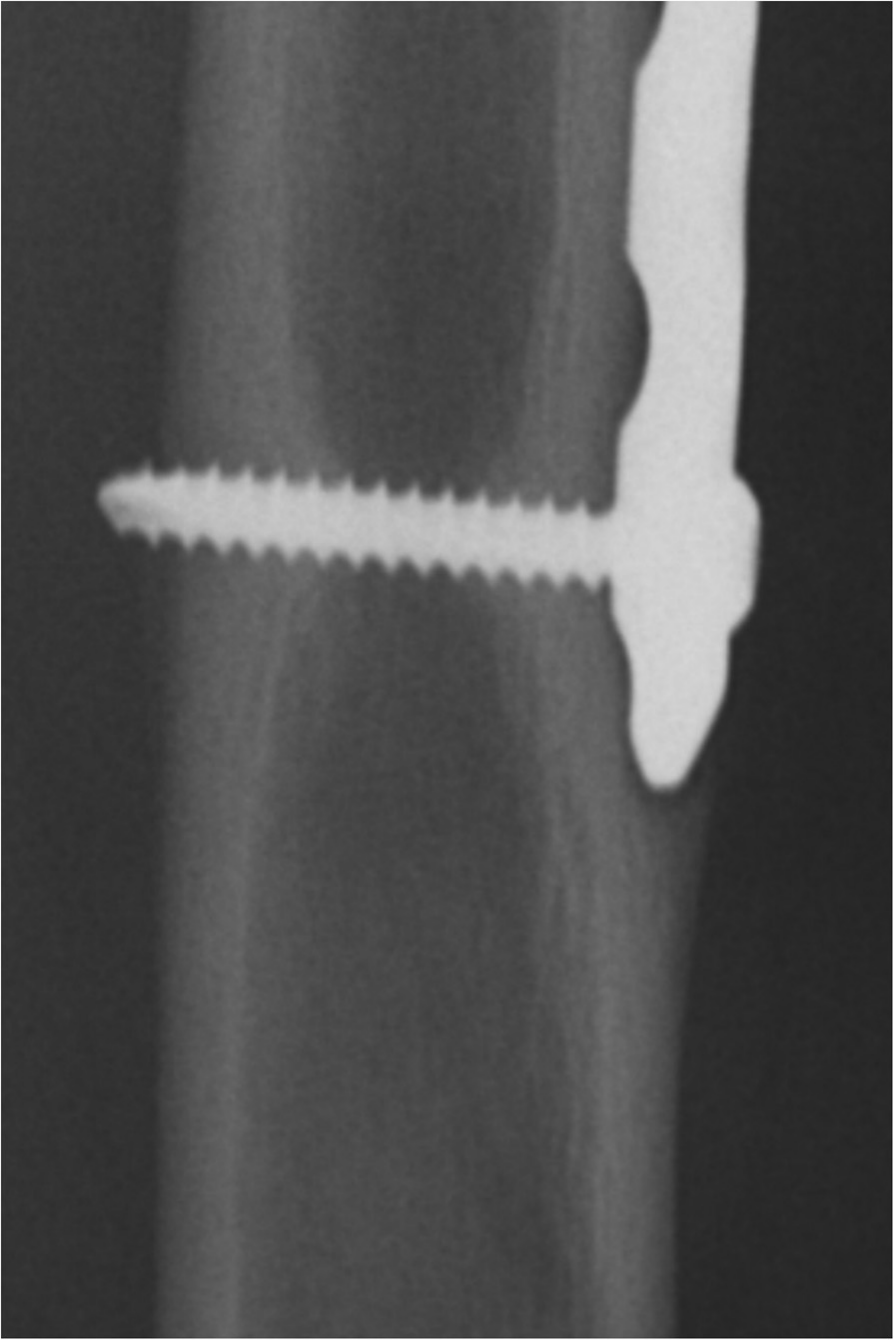
Bone beginning to grow over distal end of plate after osteosynthesis for pediatric femoral fracture.

**Figure 2:**
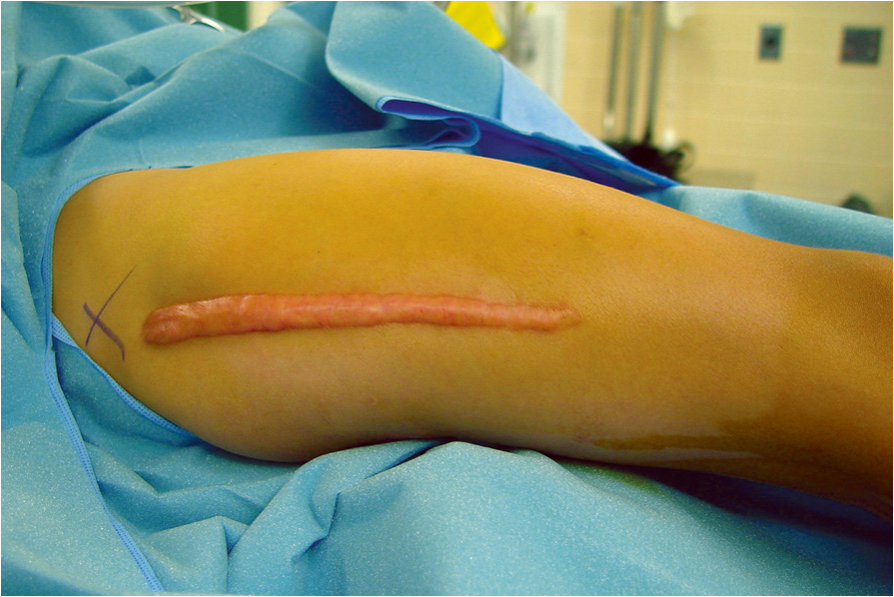
Cosmetically disadvantageous keloid formation after open approach to the femur.

A controversy exists regarding routine implant removal in children [16]. Routine removal in children was justified based on potential future risks, and many institutions offer routine removal of implants to skeletally immature patients [17-19]. Resulting thereof, 60% to 90% of all implant removals in children are performed electively [17,20,21]. Implant removal is not without complications or consequences [14,22-25]. The problem with submuscular insertion especially with titanium implants and pediatric patients is that fibrous and osseous ingrowth to the implant interferes with implant removal [26] (Figure 1). This may lead surgeons to use more extensive approaches for implant removal. Noticeably wider and cosmetically disadvantageous scars after implant removal compared to the initial approach have been reported (Figure 2). In one study, 3.6% of the patients had asked to see a plastic surgeon [27]. Some innovative techniques have been described to remove pelvic and spinal implants via minimally invasive techniques [28,29]. To our knowledge, no technique for minimally invasive implant removal on long bones has been described. Routine hardware removal is offered most often to pediatric patients [17]. Therefore, we have chosen purposefully a pediatric study population.

## Methods

### Technique

Final radiographs are preoperatively evaluated and confirmed for fracture healing, manufacturer type, and screw (standard versus locked) position. Appropriate screwdrivers are confirmed. The lower extremity is placed on a radiolucent table. The fluoroscopic imaging unit is brought in from the contralateral side of the table from the implant. Therefore, laterally applied femoral or tibial implants have the fluoroscope imaging coming from the contralateral side while medially applied tibial plates have the fluoroscopic unit on the ipsilateral side. Screw positions with the incisions and the fluoroscopic unit were confirmed. With reduced fractures and swelling after healing, the incisions may not line up perfectly with the screw sites. Small incisions corresponding to the screw sites were made. The limb was rotated so that the screw heads and implant are perpendicular to the imaging unit to improve visualization and triangulation of the screwdriver head. Once the screwdriver head is adjacent to the screw head, the anterior and posterior aspect of the plate was palpated with the screwdriver head to insure positioning in the sagittal plane. When the screwdriver is positioned appropriately in all planes (axial, coronal, and sagittal), the screwdriver head was tapped gently with a mallet while carefully reversing the screwdriver head (similar to a jeweler). Once initially seeded into the screw head, the screwdriver was further tapped until placed deep into the concavity of the screw head. Utilizing the fluoroscopic magnification will improve visualization and confirm positioning. The screw was reversed from the plate while applying pressure on the screwdriver head to avoid losing contact. Once the screw tip is within the near cortex, the screw head was grasped with a hemostat or needle driver. The screw was fully removed with a coordinated effort of gentle pressure from the screwdriver and axial removal from the hemostat or needle driver.

**Figure 3:**
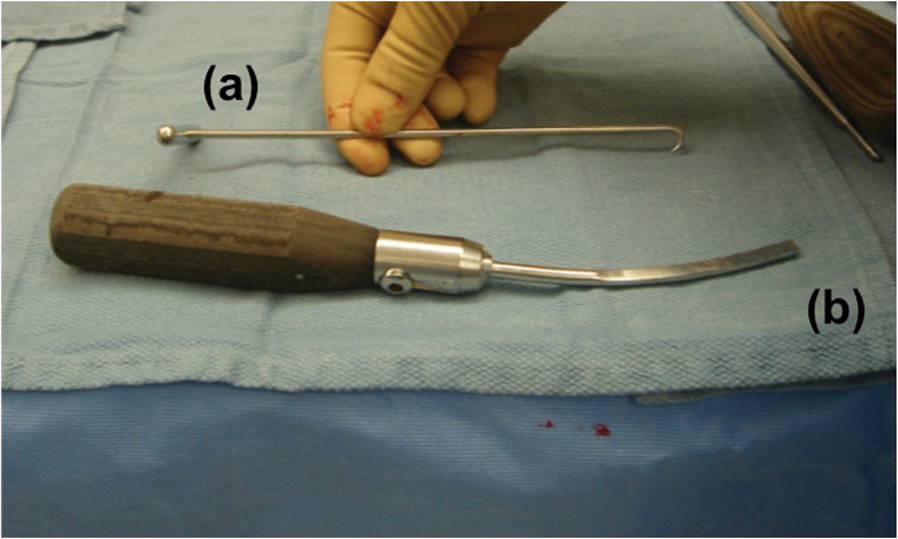
Shoulder hook (a) and bone tamp (b) for percutaneous implant removal.

**Figure 4:**
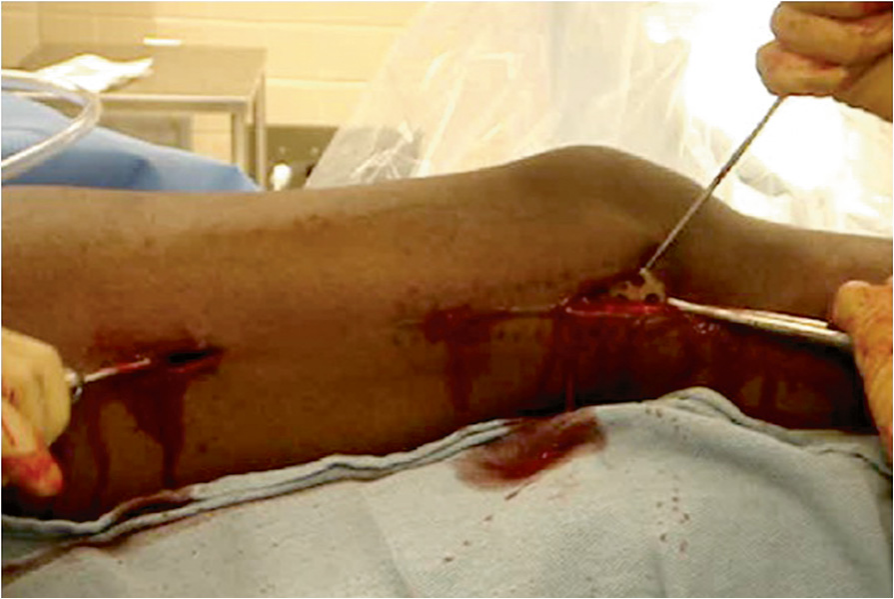
Pushing and pulling in a combined effort removes the implant with minimal soft tissue damage.

Once all the screws are removed, the incision that was initially created was opened to insert the plate. The soft tissue attachments from the end of the plate were cleared off. The plate was gently elevated off the bone with a Cobb elevator. The shoulder hook (Zimmer Inc., Warsaw, IN, USA) (Figure 3 (a)) was slipped under the plate and through a screw hole. Through a screw hole site and incision along the other end of the plate, the curved bone tamp (Synthes, Paoli, PA, USA) was percutaneously inserted (Figure 3 (b)). With a coordinated effort, the implant was gently pulled with the shoulder hook and pushed with the bone tamp (Figure 4). Hitting the angled tamp with a mallet is usually required for release of the fibrous and osseous ingrowths. The plate will usually have an initial release, which is related to the plastic deformation of the fibrous tissue. Once the plate escapes the release from the tissues, it will move quickly and with ease. To avoid the plate from scratching the skin upon removal, the shoulder hook was kept angled slightly upward. If a deep incision is present from obesity or muscularity, the Cobb elevator was kept under the plate to protect the skin and direct the plate removal. To avoid the plate to quickly escape the control of the shoulder hook upon removal, the shoulder hook was gently but not aggressively pulled on.

**Figure 5:**
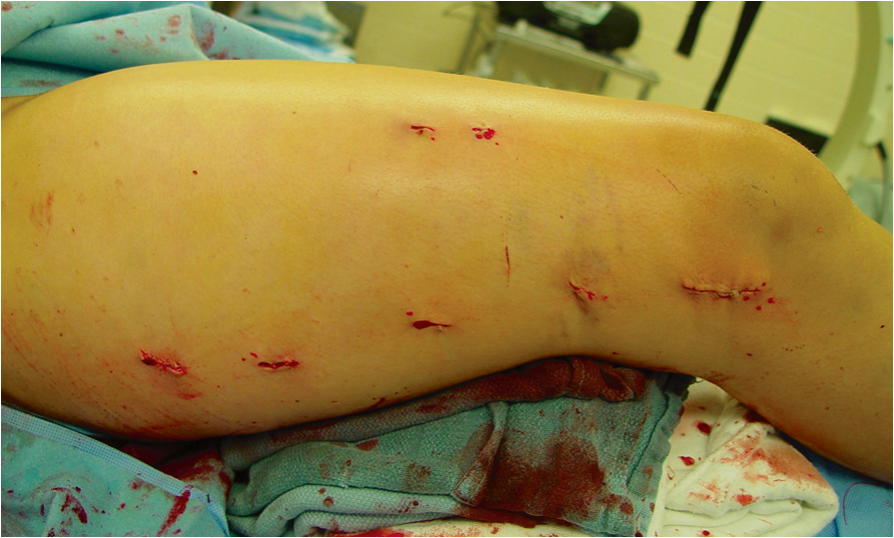
Closed incisions after percutaneous hardware removal.

Once the plate is removed and bleeding assessed, the larger incision fascia was closed with absorbable sutures. The skin was closed with nylon or Vicryl sutures (Figure 5). The incisions were infiltrated with 0.25% Marcaine based upon body weight limitations. A minimally compressive dressing was applied. Although possibly controversial, the patient was allowed weight bearing as tolerated [30-32]. Once able, the patient was allowed to return to normal activities.

### Case series

A retrospective evaluation of plate removal after submuscular plate insertion in pediatric patients was performed in one level I trauma center. All patients were initially treated, and implant removal was performed by the senior author (CBJ). Submuscular insertion techniques and patients were identified from a registry using CPT codes 27506, 27507, 27511, 27513, and 27514 of plated pediatric femoral fractures between 2005 and 2010. Patients were followed up in a single private practice.

We recorded the age at injury, the length of the plate used, and the numbers of screws placed. We recorded the time from plate insertion to plate removal. Charts were reviewed to determine the stated reason for removal. Radiographs were reviewed to determine any bony overgrowth. Operative records and office notes were reviewed to determine any noted complications during removal and postoperatively. Ethical standards were followed in the conduct and dissemination of the study.

## Results

We identified 26 patients at our institution that underwent submuscular plating for femoral fractures during the study period. Thereof, 21 (81%) implants were removed using the described submuscular technique in all cases. Four patients were followed up in their home towns, and one patient with paraplegia did not undergo hardware removal.

The average age at the time of surgery was 8 years (range, 3 to 12 years). The documented reason for plate removal was the surgeon’s recommendation [18], beginning bone overgrowth [1], and pain or irritation [2]. The median plate length was 14 holes (range, 8 to 18 holes). The mean number of proximal as well as distal screws was 2 (2 to 3). Plates were twenty 3.5-mm locking compression plates (Synthes) and one 3.5-mm compression plate (Zimmer Inc.). The mean time from plate insertion to plate removal was 8.0 months (range, 4.0 to 26.6 months).

Because of implant removal difficulties, one patient (4.8%) required a change of the procedure to an open approach. The complication was caused by a cold welded screw head. Another patient had an anticipated broken screw, which could be retrieved completely through one of the incisions. All implants were removed completely. No postoperative complications regarding hematoma formation, wound healing problems, infections, or nerve injuries were recorded.

## Discussion

Submuscular plating of pediatric femoral fractures has become a useful treatment option [33,34]. Multiple reasons, fears, and complaints of both patients and orthopedic surgeons result in the removal of previously implanted fixation devices. To avoid complete osseous integration of the implant, surgeons recommend removal of all pediatric implants used for fixation of long bone fractures [16]. Concern has been voiced regarding a more invasive and open approach for implant removal than for fracture fixation affecting pain, bleeding, healing, cosmesis, and patient satisfaction [27].

In our attempt to avoid extensive approaches for implant removal procedures after initially submuscularly inserted plates, we found this novel ‘push-pull’ technique an extremely useful tool. Open approaches with a ‘second hit’ on soft tissues and additional scarring were avoided in 92% of our patient population. Additionally, no complications regarding hematoma formation, wound healing problems, infections, or nerve injuries were noted. In performing the procedure correctly, careful tapping of the bone tamp with a mallet and coordinated pulling of the shoulder hook to guide plate removal avoid forceful removal with a clamp or hook. Therefore, we consider the procedure as safe and effective when performed for the right indication.

Indications for submuscular plate removal are well-healed fractures without complete osseous overgrowth of the implant void of overlying neurovascular structures. Therefore, lateral femoral, lateral proximal tibial, and medial tibial areas are well suited for this technique. The distal lateral tibial pilon could be removed with this technique but with caution to avoid injury to the overlying anterior tibial artery and nerve upon removal. The humerus and forearm have many crossing neurovascular structures; therefore, percutaneous plate removal should be discouraged. Even though not personally experienced, percutaneous plate removal in settings of osseous overgrowth should not be attempted [9]. Even with open techniques, plate removal can be problematic with laborious removal of all osseous ingrowth and overgrowth. Screw-related complications are uncommon and not predictable. With previously diagnosed broken screws, stripped screw heads, or cross-threaded locked screw heads, wider incisions or open techniques of successful plate removal should be encouraged. If the screwdriver will not seat in the screw head, an incision will be made to expose the screw head, the debris will be removed within the screw head, and the osseous material around the screw head and within the screw hole will be removed to avoid screw head stripping, which necessitates a larger approach and possibly inability to remove the plate without more invasive and aggressive methods. If the screw head is cross-threaded and cold worked into the threaded screw hole, the head may need to be drilled out, the plate cut with a diamond-tipped wheel leaving metal debris in the soft tissues, or the plate pulled off the osteopenic bone [26]. With friable skin or skin conditions, expanding the incisional length will avoid ripping the skin edges upon plate removal or bone tamp manipulation.

Although not as efficient, the surgeon can successfully remove the plate with alternative devices. Although not small and as easily inserted, a bone hook can be substituted for a shoulder hook. Since the shoulder hook has a ‘T’-shaped end, any substitution should allow for rotational control of the device. Also, a traditional bone tamp may be utilized but must be able to fit within the screw hole shape and have an incision large enough for angled insertion. To diminish the risk of bleeding or creating injury to the bone or soft tissue, sharp instruments for pushing such as a periosteal elevator or osteotome were avoided. The smaller sharp instrument may inadvertently slip through the screw hole creating a fracture in the bone or past point distally outside the screw hole injuring the muscle or possibly a vessel.

We acknowledge the limitations of our study. The major limitation of this study was its retrospective design and the small number of patients. Additionally, patient satisfaction was not addressed utilizing standardized questionnaires. The surgical outcome or complications were not compared to a contemporaneous control group.

This study is a structured description of a technical trick in combination with a single-center case series. Therefore, this study has the potential to reflect technical difficulties and complication incidence.

## Conclusion

Minimally invasive removal of long bone plating in pediatric patients can be successfully performed utilizing the described push-pull technique. Indications should exclude anatomic sites endangering neurovascular structures and local difficulties including bone overgrowth and broken or striped screws.

## Competing interests

The authors declare that they have no competing interests.

## Authors’ contributions

MFH participated in the conception and design of the study, performed the data acquisition, participated in the statistical analysis, and drafted the manuscript. CBJ participated in the conception and design of the study, provided administrative support, carried out the critical revision of the manuscript, and supervised the study. JG participated in the data acquisition. All authors read and approved the final manuscript.
